# In silico and empirical evaluation of twelve metabarcoding primer sets for insectivorous diet analyses

**DOI:** 10.1002/ece3.6362

**Published:** 2020-05-21

**Authors:** Orianne Tournayre, Maxime Leuchtmann, Ondine Filippi‐Codaccioni, Marine Trillat, Sylvain Piry, Dominique Pontier, Nathalie Charbonnel, Maxime Galan

**Affiliations:** ^1^ CBGP INRAE CIRAD IRD Montpellier SupAgro Université de Montpellier Montpellier France; ^2^ Nature Environnement 17 Surgères France; ^3^ LabEx ECOFECT “Ecoevolutionary Dynamics of Infectious Diseases Université de Lyon Lyon France; ^4^ CNRS Laboratoire de Biométrie et Biologie Évolutive UMR5558 Université de Lyon Université Lyon 1 Villeurbanne France

**Keywords:** arthropod, bat, environmental DNA, high‐throughput sequencing, predator feeding

## Abstract

During the most recent decade, environmental DNA metabarcoding approaches have been both developed and improved to minimize the biological and technical biases in these protocols. However, challenges remain, notably those relating to primer design. In the current study, we comprehensively assessed the performance of ten COI and two 16S primer pairs for eDNA metabarcoding, including novel and previously published primers. We used a combined approach of in silico, in vivo‐mock community (33 arthropod taxa from 16 orders), and guano‐based analyses to identify primer sets that would maximize arthropod detection and taxonomic identification, successfully identify the predator (bat) species, and minimize the time and financial costs of the experiment. We focused on two insectivorous bat species that live together in mixed colonies: the greater horseshoe bat (*Rhinolophus ferrumequinum*) and Geoffroy's bat (*Myotis emarginatus*). We found that primer degeneracy is the main factor that influences arthropod detection in silico and mock community analyses, while amplicon length is critical for the detection of arthropods from degraded DNA samples. Our guano‐based results highlight the importance of detecting and identifying both predator and prey, as guano samples can be contaminated by other insectivorous species. Moreover, we demonstrate that amplifying bat DNA does not reduce the primers' capacity to detect arthropods. We therefore recommend the simultaneous identification of predator and prey. Finally, our results suggest that up to one‐third of prey occurrences may be unreliable and are probably not of primary interest in diet studies, which may decrease the relevance of combining several primer sets instead of using a single efficient one. In conclusion, this study provides a pragmatic framework for eDNA primer selection with respect to scientific and methodological constraints.

## Graphical abstract

We applied complementary approaches to identify primer characteristics and primer sets that enable to describe the diet of two sympatric bat species, by maximizing arthropod detection and taxonomic identification, while identifying bat species and minimizing labor time and cost. These approaches included in silico investigations and in vivo analyses of mock communities including arthropod taxa from 16 arthropod orders and bat guano samples. We confirm that the level of primer degeneracy is the main factor influencing arthropod detection for in silico and mock community analyses, while the amplicon length is critical for the detection of arthropods from degraded DNA samples. We show that amplifying bat DNA does not affect the primers' capacity to detect arthropods. As such, our results advocate for the need to identify simultaneously predator and arthropod prey, as fecal samples can be contaminated (here by different insectivorous species).

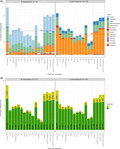

## 1 INTRODUCTION

The genetic analysis of environmental samples such as soil, water, or feces, known as environmental DNA (eDNA) metabarcoding, is a rapid and cost‐effective tool for the study of species that are difficult to detect or monitor (Bohmann et al., 2014). This approach allows the simultaneous identification of multiple taxa in environmental samples, bypassing the need to isolate organisms prior to identification (Clare, 2014; Taberlet, Coissac, Hajibabaei, & Rieseberg, 2012). eDNA metabarcoding is of particular interest in dietary analysis of rare or elusive species, and this approach has now been applied to a large spectrum of organisms (Clare, Fraser, Braid, Fenton, & Hebert, 2009; Corse et al., 2017; Kartzinel & Pringle, 2015; Rytkönen et al., 2019; Shehzad et al., 2012). Compared to the traditional microscopic study of undigested fragments in fecal remains, eDNA metabarcoding has three key advantages for dietary analysis: (a) a finer resolution (potentially to the species level), (b) the simultaneous processing and sequencing of several hundred samples, and (c) the detection of species that could not be detected using previous techniques such as visual recognition of morphological features (e.g., soft‐bodied species; Galan et al., 2018; Nielsen, Clare, Hayden, Brett, & Kratina, 2018). However, eDNA metabarcoding is subject to many biological and technical biases in each step of the process, including fieldwork, laboratory analysis, and bioinformatics. Inappropriate sampling and storage conditions, contaminations, PCR inhibitors, PCR stochasticity, and chimera formation are common biases influencing the reliability of results (for a review, see Alberdi, Aizpurua, et al., 2019; Lindahl et al., 2013). Many methodological improvements have been made to limit some of these biases and to introduce best‐practice guidelines for metabarcoding protocols, such as the systematic inclusion of technical replicates and negative controls (Alberdi, Aizpurua, Gilbert, & Bohmann, 2018; Corse et al., 2017; Elbrecht & Steinke, 2019; Galan et al., 2016; Mata et al., 2018).

Another major issue in eDNA metabarcoding protocols that still needs to be solved is the selection of appropriate primer set(s), although this issue has received increased attention in recent years (Elbrecht et al., 2019; Op De Beeck et al., 2014; Piñol, Senar, & Symondson, 2019). This is a crucial choice, as the primers must be suitable for all of the taxa actually present in the environmental sample in order to avoid missing unexpected items (Taberlet, Coissac, Pompanon, Brochmann, & Willerslev, 2012). The DNA fragment amplified by the primers must be variable enough to discriminate close species, but also needs to be abundantly referenced in public sequence databases for successful taxonomic identification based on the sequences generated (Elbrecht et al., 2016). Among the various mitochondrial genes used in animal metabarcoding, such as the 12S rRNA, 16S rRNA, and cytochrome *b* genes (Hänfling et al., 2016; Riaz et al., 2011; Santas, Persaud, Wolfe, & Bauman, 2013), the cytochrome c oxidase I (COI) gene is the most widely used as it fulfills the above criteria the best (Andújar, Arribas, Yu, Vogler, & Emerson, 2018; Hebert, Cywinska, Ball, & deWaard, 2003). Formerly, DNA barcoding approaches targeted the “Folmer region” of COI which is 658 base pairs (bp) long. This is too long for efficient sequencing using the second generation of high‐throughput sequencing (HTS) platforms (Lear et al., 2018). Moreover, eDNA is often highly degraded, due to the digestion process (in the case of fecal samples) or to environmental exposure of the samples (i.e., rain, sunlight; Oehm, Juen, Nagiller, Neuhauser, & Traugott, 2011). Such issues render the use of the entire “Folmer region” COI sequence impracticable (Deagle, Eveson, & Jarman, 2006). “Mini COI barcodes” (i.e., with a targeted region range < 200 bp; Hajibabaei et al., 2006; Pompanon et al., 2012) are therefore more commonly used in eDNA metabarcoding analyses. However, the paucity of conserved regions in the COI sequence can complicate universal primer design (Deagle, Jarman, Coissac, Pompanon, & Taberlet, 2014). Thus, some authors have argued for the joint use of several COI primer sets (e.g., Corse et al., 2019; Esnaola, Arrizabalaga‐Escudero, González‐Esteban, Elosegi, & Aihartza, 2018) or for the combination of mitochondrial 16S rRNA and COI primer sets (e.g., Alberdi et al., 2018; Bohmann et al., 2018). This combined approach should improve the taxonomic coverage of prey items. For example, Esnaola et al. (2018) have shown that the combination of the Zeale, Butlin, Barker, Lees, and Jones (2011) and Gillet et al. (2015) primer sets enabled the detection of 37.2% more species than when using Gillet's primer set alone. However, the combination of several primer sets greatly increases both the financial cost and the duration of laboratory and bioinformatics analyses of metabarcoding studies. In this context, the use of a single highly degenerate primer set would be preferred, and some studies have already highlighted the efficiency of this approach (Elbrecht & Leese, 2017; Galan et al., 2018; Vamos, Elbrecht, & Leese, 2017). Degenerate bases are used in primer sequences to avoid mismatches at variable positions between the different targeted taxa. In theory, this process should enable the amplification and identification of many different taxa using a unique primer set in a single PCR. However, degenerate primers have to be carefully designed because high levels of degeneracy can lead to high rates of nontarget amplification (Innis, Gelfand, Sninsky, & White, 2012). No consensus on primer choice has yet been reached in eDNA metabarcoding, and at present, a multitude of primer sets exist for the COI and 16S genes that differ in their target length, degeneracy levels, and position within the genes.

Designing robust metabarcoding protocols for the study of insectivorous bat diets is still an important issue. Such molecular analyses are critical because the direct observation of bat feeding is virtually impossible. The morphological identification of prey remains in guano rarely provides resolution at the genus or species level and can be highly time‐consuming. Finally, many bat species are endangered and highly protected such that invasive methods cannot be used to carry out diet surveys. Metabarcoding analysis of the DNA contained in bat guano has thus been developed (a) to deepen our understanding of bat ecology (Arrizabalaga‐Escudero et al., 2015; Clare, Symondson, Broders, et al., 2014), (b) to highlight the potential ecosystem services provided by bats as pest suppressors (Aizpurua et al., 2018; Maslo et al., 2017; Wray et al., 2018), and ultimately, (c) to set up effective conservation strategies (Arrizabalaga‐Escudero et al., 2015). However, most of these studies have used specific arthropod primer sets (but see Galan et al., 2018; Jusino et al., 2018; Wray et al., 2018) or specific bat primer sets (Walker, Williamson, Sanchez, Sobek, & Chambers, 2016). While working with guano collected from roost sites, a potential problem arises from the fact that several bat species may roost in the same sites. Because guano is not easily distinguishable between bat species (Ware, Garrod, Macdonald, & Allaby, 2019), it is critical to identify bat species to avoid misassigning prey to the wrong bat species and also to discard guano samples that could be contaminated with excreta from other bat species. To this end, Galan et al. (2018) previously optimized a metabarcoding approach to simultaneously identify bat species and their prey, instead of using different primer sets and methodologies to identify bat species on the one hand and arthropods on the other (e.g., Bohmann et al., 2011; Van den Bussche et al., 2016). As the amplification of bat DNA may reduce the sensitivity of prey DNA detection (Pompanon et al., 2012), it is important to find a trade‐off between the success of an exhaustive prey amplification and bat species identification.

In this study, we followed a multicriteria assessment approach to compare the performance of several sets of primers for use in insectivorous bat dietary analyses. We compared statistically different primer sets to identify the characteristics (primer degeneracy, amplicon length, DNA degradation) that would maximize the accuracy of arthropod detection and identification, while minimizing the time and financial cost of laboratory work. We also compared the capacity of primer sets to provide identification of bat species without over amplifying bat DNA. We focused on the greater horseshoe bat (*Rhinolophus ferrumequinum*) and Geoffroy's bat (*Myotis emarginatus*) as they often share maternity roosts during summer but have contrasting diets (Goiti et al., 2011; Jones, 1990). Following the recent recommendations of Elbrecht et al. (2019), we carried out a complete primer assessment based on three steps. We first made an in silico comparison of primer sets, using hundreds of thousands of arthropod sequences available in public databases. This step enabled us to evaluate primer efficiency to detect a wide taxonomic range of arthropods, independently of both the quality of samples and the effects of laboratory procedures (extraction, PCR, etc.). We then performed an in vivo comparison of primer sets using two mock communities (MC)—one containing arthropod DNA only and the other one containing both arthropod and bat DNA. This step enabled us to evaluate how efficiently primer sets detect a wide taxonomic range of known arthropod taxa, and to assess whether the presence of bat DNA would affect the efficiency of prey detection. Lastly, we made an in vivo comparison of primer sets using guano samples collected from two insectivorous bat species. This step allowed us to compare the efficiency of primer sets when amplifying degraded arthropod DNA; a common scenario for guano samples collected from roost sites.

## 2 MATERIALS AND METHODS

### 2.1 In silico evaluation

**FIGURE 1:**
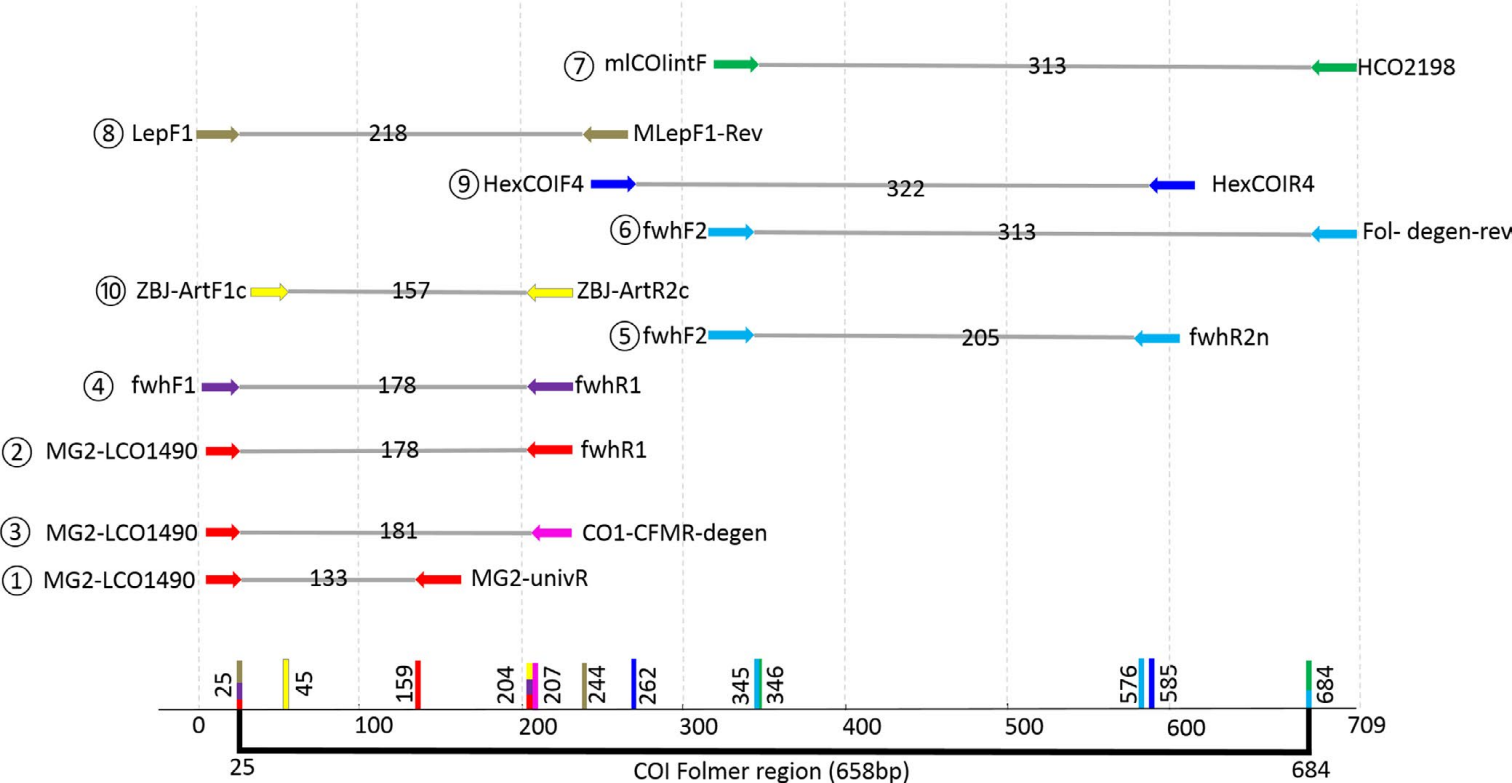
Visual representation of DNA barcode lengths and primer positions on the COI gene. Primer sets are represented by a number (see Table 1) and colored arrows, with each color representing a unique primer set. For each primer set, the number on the gray line corresponds to the amplicon length excluding primers. This information is collated at the bottom of the figure, with the whole 658‐bp COI Folmer region represented in black and the 3′ position of each primer indicated with traits of its respective color

First, we selected primers that are commonly used in arthropod metabarcoding studies (Table 1, Figure 1). Six of the selected primer sets amplified fragments within the Folmer region of the COI gene, and one set amplified a region in the 16S gene. We favored primer sets which amplified short fragments (between 133 and 218 bp excluding primers), but we also looked at longer fragments (between 313 and 322 bp excluding primers) to evaluate the potential effect of amplicon length on prey detection and identification in bat guano. Then, we designed five new combinations of primers (four for COI and one for 16S) based on the seven primer sets described above. We increased the base degeneracy level of the COI reverse primer described in Jusino et al. (2018) and of the 16S primers from Epp et al. (2012), to improve primer binding during PCR amplification. Although some studies have advocated for the combined use of 16S and COI primers (Alberdi et al., 2018; Bohmann et al., 2018; Kaunisto, Roslin, Sääksjärvi, & Vesterinen, 2017), we only included two 16S primer sets in this study, as 16S does not currently result in significant gains in taxonomic discovery for dietary studies due to a lack of local reference sequences for this region (Clarke, Soubrier, Weyrich, & Cooper, 2014; Elbrecht et al., 2016; Marquina, Esparza‐Salas, Roslin, & Ronquist, 2019). We did not include the COI primer sets proposed by Shokralla et al. (2015) which span the entire COI barcode region. Indeed, previous studies have shown that these primers do not perform efficiently with degraded DNA, probably because of the length of the fragments (respectively 325 and 418 bp excluding primers) (Haran et al., 2018). Therefore, they were not considered appropriate for the metabarcoding analyses of guano samples.

**TABLE 1:** Primer set information. Bases in bold indicate the bases which were modified (degenerated or corrected) in this study

	Custom name	Region	Primer names	Sequence 5′–3′	Targeted region length	Amplicon length	References
①	MG2	COI	F: MG2‐LCO1490	TCHAC**H**AAYCAYAARGAYATYGG	133	185	This study, based on Gillet et al. (2015) and Galan et al. (2018)
			R: MG2‐univ‐R	AC**Y**AT**R**AA**RA**A**R**A**TY**ATDAY**R**AADGCRTG			
②	MG2fwh	COI	F: MG2‐LCO1490	TCHAC**H**AAYCAYAARGAYATYGG	178	221	This study, based on Galan et al. (2018)
			R: fwhR1	ARTCARTTWCCRAAHCCHCC			Vamos et al. (2017)
③	MG2ANML‐degen	COI	F: MG2‐LCO1490	TCHAC**H**AAYCAYAARGAYATYGG	181	224	This study, based on Galan et al. (2018)
			R: CO1‐CFMR‐degen	A**YN**A**R**TCA**R**TT**H**CC**R**AA**H**CC			This study, based on Jusino et al. (2018)
④	fwh1	COI	F: fwhF1	YTCHACWAAYCAYAARGAYATYGG	178	222	Vamos et al. (2017)
			R: fwhR1	ARTCARTTWCCRAAHCCHCC			
⑤	fwh2	COI	F: fwhF2	GGDACWGGWTGAACWGTWTAYCCHCC	205	254	Vamos et al. (2017)
			R: fwhR2n	GTRATWGCHCCDGCTARWACWGG			
⑥	fwhFol	COI	F: fwhF2	GGDACWGGWTGAACWGTWTAYCCHCC	313	365	Vamos et al. (2017)
			R: Fol‐degen‐rev	TANACYTCNGGRTGNCCRAARAAYCA			Yu et al. (2012)
⑦	mlHCO	COI	F: mlCOIintF	GWACWGGWTGAACWGTWTAYCCYCC	313	364	Leray et al. (2013)
			R: HCO2198	TAAACTTCAGGGTGACCAAAAAATCA			Folmer, Hoeh, Black, and Vrijenhoek (1994)
⑧	Lep1	COI	F: LepF1	ATTCAACCAATCATAAAGATATTGG	218	265	Brandon‐Mong et al. (2015)
			R: MLepF1‐rev	CGTGGAAAWGCTATATCWGGTG			
⑨	HEX	COI	F: HexCOIF4	HCCHGAYATRGCHTTYCC	322	358	Marquina, Andersson, and Ronquist (2019)
			R: HexCOIR4	TATDGTRATDGCHCCNGC			
⑩	Zeale	COI	F: ZBJ‐ArtF1c	AGATATTGGAACWTTATATTTTATTTTTGG	157	211	Zeale et al, (2011)
			R: ZBJ‐ArtR2c	WACTAATCAATTWCCAAATCCTCC			
⑪	Epp‐degen	16S	F: Epp‐degen‐F	T**R**C**W**AAGGTAGCATAATMA**H**T**W**G	106	148	This study, based on Epp et al. (2012)
			R: Epp‐degen‐R	T**YW**AT**R**GGGTCTT**V**T**Y**GTC			
⑫	Epp	16S	F: Coleop_16Sc	TGCAAAGGTAGCATAATMATTAG	106	148	Epp et al. (2012)
			R: Coleop_16Sd	TCCATAGGGTCTTCTCGTC			

**FIGURE 2:**
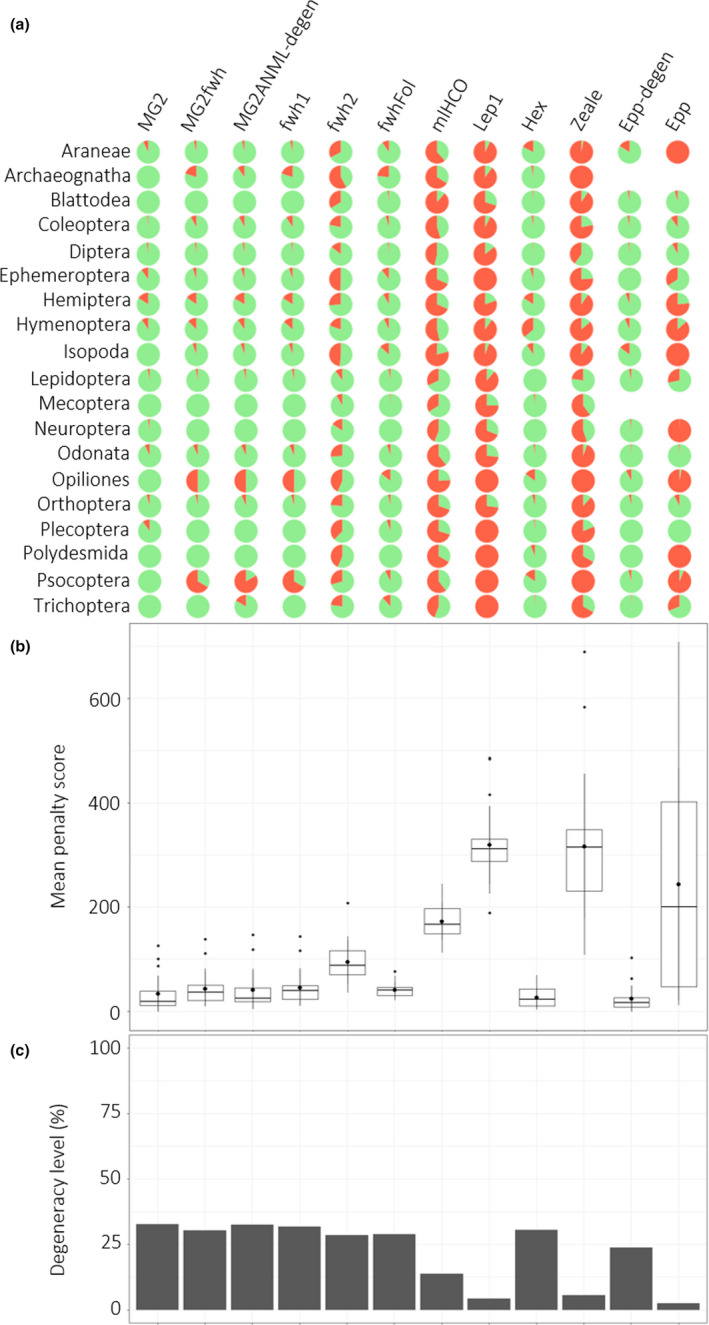
In silico evaluation of arthropod orders represented by at least 100 OTUs using *PrimerMiner*. (a) Primer set performance is shown for each order using pie charts, with green and red colors representing success and failure, respectively, of in silico amplification. Success of amplification corresponded to *PrimerMiner* mean penalty score < 120 and amplification failure to a mean penalty score ≥ 120. (b) Boxplots of the median of *PrimerMiner* mean penalty scores over all arthropod orders and for each primer set, with mean values represented by a circle within boxplots. (c) Percentage of degeneracy level of each primer set

Thus, our aim was to compare the efficiency of 12 primer sets. We used the R package *PrimerMiner* (Elbrecht & Leese, 2016) on 21 arthropod orders including potential bat prey (Figure 2). We clustered 4,259,845 sequences from the BOLD database into 327,412 COI OTUs (operational taxonomic units) and 83,651 sequences from the NCBI database into 25,505 16S OTUs. COI and 16S sequences were aligned separately using MAFFT v7.017 (Katoh, Misawa, Kuma, & Miyata, 2002) as implemented in GENEIOUS v8.1.7 (Kearse et al., 2012). The consensus sequence alignment for each order of arthropods was visualized using *PrimerMiner* which facilitates the identification of suitable primer binding sites. All primer sets (previously published and redesigned) are detailed in Table 1. They varied in target region length and degeneration level (Figure 1).

We used the mean penalty score per arthropod order provided in *PrimerMiner*. This score is calculated as a mismatch scoring that includes the adjacency, position, and type of mismatch between primers and template sequences. Primer evaluation was conducted only for arthropod orders represented by at least 100 OTUs, as recommended by Elbrecht and Leese (2016). This threshold enabled us to capture a large portion of the variability potentially existing at primer binding sites and to limit potential biases resulting from the presence of only few sequences when evaluating the match/mismatch. We compared primer sets by combining the penalty scores from both the forward and reverse primers. We then used these scores to determine whether the arthropod OTUs would theoretically be successfully amplified using the default value (success: penalty score < 120).

### 2.2 In vivo evaluation

#### 2.2.1 Mock community: sample collection and preparation

Briefly, we collected 33 arthropod individuals representing 16 orders with two taxa per order except for Opiliones, Dermaptera, Isopoda, Psocodea, Julida, Polyxenida, and Raphidioptera (which had one taxa per order). They were captured alive in May and June 2019 in the South of France and then immediately frozen at −20°C to avoid DNA degradation. Bat DNA was extracted from tissue samples. Greater horseshoe bats (*R. ferrumequinum*) were captured in Western France in 2016, and a piece of patagium (wing membrane) was collected using a 3‐mm‐diameter biopsy punch, after which bats were released. These bat samples were preserved in 95% ethanol solution at 4°C until DNA extraction and pooling.

The DNA of arthropods and bats was extracted using the EZ‐10 Spin Column Genomic DNA Miniprep Kit for Animal (BioBasic) following the manufacturer's instructions. DNA extractions were normalized to 7 ng/µl after Qubit fluorometer quantification (Invitrogen). The integrity of DNA was evaluated by electrophoresis on a 1.5% agarose gel.

We built two versions of the same mock community (MC) of 33 different arthropod taxa. The first one included the 33 arthropod DNA extracts mixed in equimolar proportions (MC_arthr_). The second one included 50% of this mock community and 50% of bat DNA (MC_arthr+bat_). The *R. ferrumequinum* DNA used in the mock community was a pool of DNA extractions representing 18 individuals of the same colony. This design enabled us to limit the use of DNA per individual and to preserve this material for future studies.

We used the same DNA extractions of bats and arthropods to build reference sequences for the COI and 16S genes. Normalized DNA was amplified and sequenced for each individual to provide reference sequences for each gene. As the ten COI primers span almost all of the 658‐bp COI Folmer region, we sequenced this region using Sanger technology as described in Sow et al. (2018). We sequenced the 106‐bp region targeted by the two 16S minibarcodes using the Epp‐degen primer set and MiSeq sequencing technology for each DNA extraction independently, following the same protocol used for the analysis of the mock and guano samples, as described below.

#### 2.2.2 Guano samples: collection and preparation

We collected 22 fecal pellets from each of five mixed maternity colonies of the greater horseshoe bat and Geoffroy's bat in Western France in June 2018. Each fecal pellet was retrieved from paper plates which had been left on the ground beneath the colony for 10 days. Single‐use forceps were used to collect pellets to avoid contaminations between samples. Paper plates were renewed on each sampling date to avoid contaminations. Samples were stored at −20°C until DNA extraction.

Briefly, guano samples were frozen at −80°C and then bead‐beaten for 2 × 30 s at 30 Hz in a TissueLyser (Qiagen) using a 5‐mm stainless steel bead. DNA was extracted using the NucleoSpin 8 Plant II kit (Macherey‐Nagel) with the slight modifications recommended in Zarzoso‐Lacoste et al. (2018).

#### 2.2.3 PCR and library construction

The PCR conditions can greatly influence the performance of primer sets (Jusino et al., 2018). Thus, we applied the same PCR program for all primer sets and used a low annealing temperature (45°C) as recommended in recent studies (Elbrecht et al., 2019; Jusino et al., 2018). We validated this program by checking the quality of PCR amplifications on the mock communities for the 12 primer sets.

We made four versions of each primer by adding a 5′ heterogeneity spacer of 0–3 bp to the primer sequence. This increased the diversity at each sequencing cycle, improved the detection of the sequencing clusters at the flowcell surface, and thus increased the quality of the reads. The four versions of each primer were mixed together before PCR.

We used the two‐step PCR protocol described in Galan et al. (2018) with slight modifications. Firstly, we increased the extension time (2 min instead of 45 s) in PCR_1_ and PCR_2_ to reduce chimera formation (Qiu et al., 2001). Secondly, for each PCR replicate multiplexed in the same run, we used a double indexing strategy as recommended in Kircher, Sawyer, and Meyer (2012) to significantly reduce the rate of read misassignment: Each 9‐bp i5 and i7 dual index was used only for one PCR sample, eliminating the problem of “leak” due to false index‐pairing (Martin, 2019). PCR_1_ was performed in 10 µl reaction volumes using 5 µl of 2× Qiagen Multiplex Kit Master Mix (Qiagen), 2.5 µl of ultrapure water, 0.5 µl of each mix of forward and reverse primers (10 µM), and 1.5 µl of DNA extract. Thermocycler conditions for PCR_1_ consisted of an initial denaturation step at 95°C for 15 min, followed by 40 cycles of denaturation at 94°C for 30 s, annealing at 45°C for 45 s, and extension at 72°C for 2 min, followed by a final extension step at 72°C for 10 min. Conditions for PCR_2_ consisted of a limited‐cycle amplification step to add multiplexing indexes i5 and i7 (nine bases each) and Illumina sequencing adapters P5 and P7 at both ends of each DNA fragment from PCR_1_. PCR_2_ was carried out in a 10 µl reaction volume using 5 µl of Qiagen Multiplex Kit Master Mix (Qiagen) and 2 µl of each indexed primer i5 and i7 (0.7 µM). Then, 2 µl of PCR_1_ product was added to each well. The PCR_2_ started by an initial denaturation step of 95°C for 15 min, followed by eight cycles of denaturation at 94°C for 40 s, annealing at 55°C for 45 s, and extension at 72°C for 2 min, followed by a final extension step at 72°C for 10 min.

We included a negative control for extraction (NC_ext_), a negative control for PCR (NC_PCR_), and a negative control for indexing (NC_index_) in each 96‐well microplate. We performed three PCR technical replicates per sample on each DNA extract. For the guano samples, we considered a sample positive for a particular taxon if at least two out of three replicates were positive, to overcome the stochasticity of the PCR in the detection of rare prey while reducing the number of putative false‐positive results (i.e., one positive replicate out of three is considered a false‐positive result; Alberdi et al., 2018).

We checked the homogeneity of amplifications between primer sets and for nonspecific amplification by electrophoresis using 3 µl of each PCR_2_ product on a 1.5% agarose gel. Then, PCR_2_ products were pooled separately for each of the 12 primer sets and put on a low‐melting agarose gel (1.25%) for excision. After electrophoresis, the excision step was used to eliminate primer dimers and nonspecific amplifications. We used the PCR Clean‐up Gel Extraction kit (Macherey‐Nagel) to purify the excised bands. The 12 pools were quantified using the KAPA library quantification kit (KAPA Biosystems) taking into account the different fragment lengths, normalized at 4nM, and pooled in equimolar concentrations before loading 14 pM and 5% of PhiX control on a MiSeq flowcell with a 500‐cycle Reagent Kit v2 (Illumina). The mock communities and the guano samples were sequenced independently on two different Illumina runs.

#### 2.2.4 Bioinformatics and taxonomic assignments

First, we used a R preprocessing script (Sow et al., 2019) to merge paired‐end sequences into contigs with FLASH v.1.2.11 (Magoč & Salzberg, 2011) and to trim primers with CUTADAPT v.1.9.1 (Martin, 2011). We then used the FROGS pipeline (“Find Rapidly OTU with Galaxy Solution”; Escudié et al., 2018) to create an abundance table for each variant. Briefly, this pipeline enabled us to (a) filter sequences by length (±20 bp from the expected length), (b) cluster the variants into operational taxonomic units (OTUs) using a maximum aggregation distance of one mutation with the SWARM algorithm (Mahé, Rognes, Quince, de Vargas, & Dunthorn, 2014), (c) remove chimeric variants using VSEARCH with de novo UCHIME method (Edgar, Haas, Clemente, Quince, & Knight, 2011; Rognes, Flouri, Nichols, Quince, & Mahé, 2016), and (d) filter by keeping only OTUs present in at least two PCR replicates.

Taxonomic assignments were carried out for each primer set, following different procedures. We used the Sanger reference sequences produced to analyze mock community results (see above). This enabled us to identify the 33 genuine arthropod sequences in the two mock communities and the genuine bat sequences. The other sequences were excluded from subsequent analyses. With regard to guano samples, we analyzed the 16S OTUs using BLASTN (Altschul, Gish, Miller, Myers, & Lipman, 1990) and the NCBI Nucleotide database (Benson, Karsch‐Mizrachi, Lipman, Ostell, & Wheeler, 2008). Taxonomic assignments of COI OTUs were made using the NCBI BLAST+ automatic affiliation tool available in the FROGS pipeline, with the Public Record Barcode Database (data related to the BOLD database, http://v3.boldsystems.org, accessed in February 2019, with maximum 1% of N).

### 2.3 Statistical analyses

#### 2.3.1 In silico data

We tested the effect of the level of primer degeneracy on mean penalty score and on theoretical amplification success. We used a Poisson and a binomial generalized linear mixed model (GLMM), respectively, with the primer set and the order as random effects.

#### 2.3.2 Mock community data

We tested the effect of amplicon length, level of primer degeneracy, and percentage of bat reads on the percentage of arthropod taxa detected using a binomial generalized linear model (GLM).

#### 2.3.3 Guano sample data

We tested the effect of amplicon length, level of primer degeneracy, total number of reads, and percentage of bat reads on the number of arthropod occurrences using a Poisson GLM. We did not work on the number of OTUs but rather on taxa occurrence. This was preferred as it enabled us to take into account the frequency of detection of a particular taxon for each primer set, and hence to minimize the importance of rare taxa (i.e., those detected in a single sample). As such, we minimized artificial inflations of OTUs diversity.

Note that we did not include the 16S primer sets in the in vivo statistical analyses (mock communities and guano samples) because of confounding factors (e.g., smaller size of the 16S reference database compared to the COI database).

#### 2.3.4 Defining a strategy to determine the best primer set(s) for the study

We developed a multicriteria table that included criteria for each step of primer set evaluation (in silico, mock community, and guano sample analyses). We provided a score for each criterion with regard to the objectives and constraints of our future metabarcoding studies.

## 3 RESULTS

### 3.1 In silico evaluation

The in silico evaluation was performed on 21 arthropod orders (Figure 2). We recovered almost fifty times more sequences for the COI (4,259,845 sequences) than for the 16S gene (83,651 sequences) using the BOLD and NCBI databases. Two orders, Dermaptera and Julida, and four orders, Archaeognatha, Dermaptera, Julida, and Mecoptera, were excluded from the COI and 16S analyses, respectively, using *PrimerMiner* due to their insufficient number of OTUs (<100).

The mean penalty scores and the theoretical amplification success varied strongly between primer sets and between arthropod orders (Figure 2). We found a significant negative effect of the level of primer degeneracy on the mean penalty score (GLMM, *p* = 1.01e−12) and a positive influence on the theoretical amplification success (GLMM, *p* = 2.88e−09) (Table 2). The highest mean penalty scores (>165) and lowest amplification success rates (mean < 50%) were observed for the primer sets exhibiting a lack of degeneracy (e.g., mlHCO, Lep1, Zeale, and Epp). For example, the mean penalty score of Epp primers was about 12 times higher than the score of its degenerate version Epp‐degen.

**TABLE 2:** Effect of the primer set characteristics on the in silico amplification success and mean penalty score, the detection of mock community taxa, and the detection of arthropod occurrences in guano samples

Analysis	Dataset	Model	Family	Response variable	Source of variation	Estimate	Standard error	*z* value	*p*	*df*	AIC
In silico	COI + 16 primer sets	GLMM	Binomial	Amplification success (%)	Intercept	−1.59523	0.56681	−2.814	0.005**	201	41,322
					Degeneracy level	0.12831	0.02161	5.938	2.88e−09***		
			Poisson	Mean penalty score	Intercept	5.89199	0.26983	21.84	<2.e−16***	201	6,322
					Degeneracy level	−0.07389	0.01036	−7.13	1.01e−12***		
Mock community (MC_arthr_)	COI primer sets only	GLM	Binomial	Percentage of arthropod taxa detected (%)	Intercept	0.530523	1.278847	0.415	0.678	6	22.047
					Amplicon length	−0.002179	0.005302	−0.411	0.681		
					Degeneracy level	0.191698	0.048013	3.993	6.54e−05***		
Mock community (MC_arthr + bat_)	COI primer sets only	GLM	Binomial	Percentage of arthropod taxa detected (%)	Intercept	0.076010	1.207762	0.063	0.950	6	27.6
					Amplicon length	0.001353	0.004865	0.278	0.781		
					Percentage of Chiroptera reads	0.101432	0.236613	0.429	0.668		
					Degeneracy level	0.135181	0.040169	3.365	0.001***		
Guano	COI primer sets only	GLM	Poisson	Number of occurrences	Intercept	4.306e+00	6.076e−01	7.086	1.38e−12***	5	78.986
					Amplicon length	−1.698e−03	7.845e−04	−2.165	0.030*		
					Number of reads	1.242e−06	5.806e−07	2.139	0.032*		
					Percentage of Chiroptera reads	−4.203e−03	2.640e−03	−1.592	0.111		
					Degeneracy level	7.954e−03	5.388e−03	1.476	0.140		
Guano	COI primer sets only without the Hex primer set	GLM	Poisson	Number of occurrences	Intercept	4.741e+00	9.169e−01	5.171	2.33e−07***	4	72.374
					Amplicon length	−2.026e−03	9.364e−04	−2.163	0.030*		
					Number of reads	7.841e−07	9.264e−07	0.846	0.397		
					Percentage of Chiroptera reads	−5.659e−03	3.502e−03	−1.616	0.106		
					Degeneracy level	7.958e−03	5.391e−03	1.476	0.140		

* *p* < .5.

** *p* < .01.

*** *p* < .001.

### 3.2 In vivo evaluation—Mock communities

#### 3.2.1 Sequencing results

DNA quality checks revealed that DNA was of high molecular weight and intact for all taxa considered, except for Hemiptera‐1 (*Uroleucon* sp.) whose DNA was degraded. Homogeneous amplification between primer sets was demonstrated by MC PCR product migration on agarose gels. The MiSeq sequencing run produced a total of 3,100,343 reads for the two MCs analyzed in this study. Removing reads with unexpected lengths excluded up to 6.3% of the reads (Hex primer set), while removing chimeras excluded up to 4.67% of the reads (fwhFol primer set) and removing sequences not shared by at least two PCR replicates excluded up to 21.64% of the reads (mlHCO primer set). The remaining reads varied from 168,815 for Hex to 284,216 for Lep1.

Note that we kept only the sequences that exhibited 100% identity with the reference Sanger sequences. These sequences represented between 3.16% and 10.03% of the OTUs in the mock communities but between 76.23% and 98.42% of the reads depending on the primer set (mean over all primer sets and mock communities = 92.53%).

#### 3.2.2 Detection of bat DNA

**FIGURE 3:**
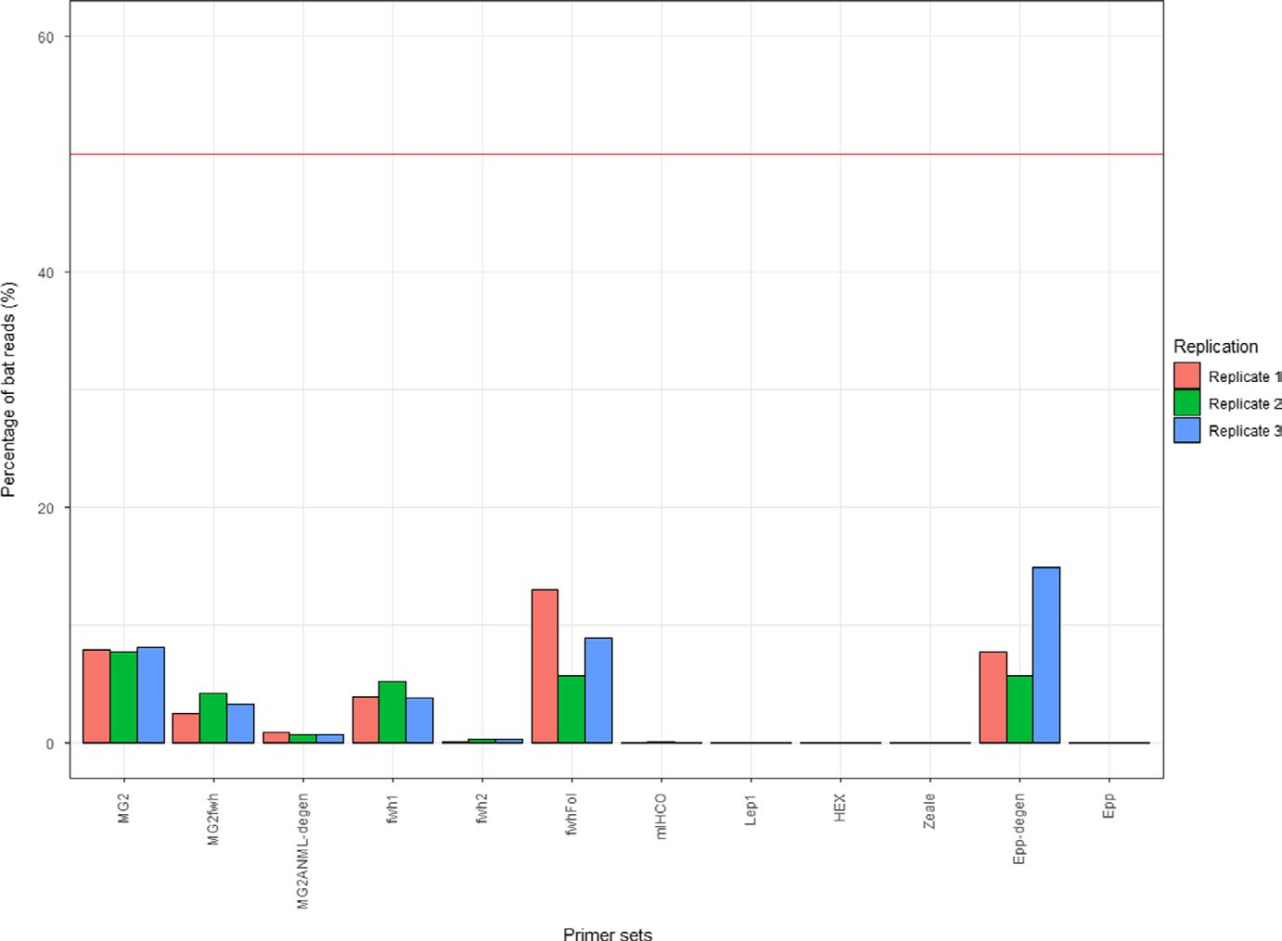
Percentage of bat reads for each primer set in the mock community MC_arthr+bat_. The red line indicates the expected value for percentage of bat reads (50%, corresponding to the DNA quantity in the DNA pool)

All primer sets amplified less bat DNA (up to 18.36% of the number of reads; replicate 2 of Epp‐degen) than the expected 50% (as arthropod and bat DNA were in equimolar proportions in the MC_arthr+bat_; Figure 3). Primers with a lack of degeneracy amplified only a few reads (Hex, mlHCO, and Epp; triplicate mean < 0.01%) or did not amplify any bat DNA at all (Zeale and Lep1). The degenerate primers fwh2 and MG2ANML‐degen amplified <1% of bat reads and fwh1 and MG2fwh <5.2%. The best primer sets for the amplification of bat DNA were Epp‐degen (triplicate mean 10.17%), fwhFol (triplicate mean 7.53%), and MG2 (triplicate mean 7.43%).

#### 3.2.3 Detection of arthropod taxa

**FIGURE 4:**
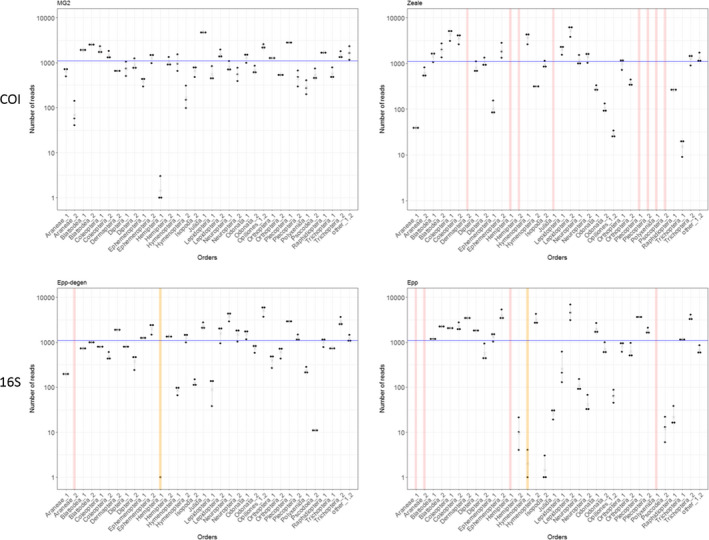
Representation on a log‐scale of the number of reads gathered for each genuine arthropod sequence with the MG2 and Zeale COI primer sets, and Epp and Epp‐degen 16S primer sets, and for the three PCR technical replicates of the mock community MC_arthr_. The blue line indicates the expected number of reads. Each dot represents a technical replicate. Yellow bars emphasize situations where only one or two PCR replicates out of three, generated reads for the taxon in question. Red bars emphasize situations where none of the PCR replicate generated reads for the taxon in question

The percentage of arthropod taxa detected in MCs varied from 67% to 100% (see Figure 4 and Figure S1). In the mock community lacking bat DNA (MC_arthr_), MG2 was the only primer set that amplified all arthropod orders in triplicate. Five other primer sets (MG2fwh, MG2ANML‐degen, fwh1, fwh2, and fwhFol) amplified all arthropod orders; however, the taxa with degraded DNA (*Uroleucon* sp. Hemiptera‐1) amplified only few reads in two replicates out of three. Hex was also among the best primer sets as it amplified all taxa except the degraded Hemiptera‐1. Hemiptera‐1 was misamplified by less than half of the primer sets (only one to three reads were recorded) and not amplified by the others. In accordance with the in silico evaluation, mlHCO, Lep1, Zeale, and Epp failed to detect an important number of taxa, and Zeale and Lep1 were the less efficient primer sets with, respectively, eight and 14 taxa unamplified or misamplified (two replicates out of three). Our results revealed a positive effect of COI primer degeneracy levels on the percentage of detected taxa in MC_arthr_ (GLM, *p* = 6.54e−05; Table 2) but no effect of the amplicon length (GLM, *p* = .681; Table 2). The influence of the degeneracy level is also illustrated by the two 16S primer sets: Epp misamplified or did not amplify five (MC_arthr_) and six taxa (MC_arthr+bat_), while its degenerated version Epp‐degen misamplified or did not amplify only two taxa of the mock communities.

In the mock community containing bat DNA (MC_arthr+bat_), four primer sets, MG2, MG2ANML‐degen, fwh2, and fwhFol, amplified all taxa in triplicate (Figure S1). The primer set fwh1 performed with reduced efficiency as it did not amplify Hemiptera‐1 at all. The two primer sets Zeale and Lep1 were again the least efficient with eight and 13 taxa that were not amplified or misamplified, respectively. Our results revealed a positive effect of the level of COI primer degeneracy on the percentage of taxa detected in MC_arthr+bat_ (GLM, *p* = .001; Table 2). We found no effect of amplicon length (GLM, *p* = .781; Table 2) or of the percentage of bat reads (GLM, *p* = .668; Table 2).

In both MCs, the primer sets that detected the smallest number of taxa (Zeale, Lep1, mlHCO, and Epp) also exhibited the largest variation of read numbers between arthropod taxa and between the observed and expected number of reads (Figure S1).

#### 3.2.4 Taxonomic identification of arthropod taxa

All COI primer sets led to identical identifications of arthropod taxa and exhibited only slight differences in taxonomic resolution. In contrast, the COI and 16S primer sets often led to different identifications and levels of taxonomic resolution. For example, identical identifications and resolutions were found for only eight taxa among the 33 arthropod taxa included in the MCs. The 16S sequences always reached a lower level of taxonomic resolution. The only exceptions were (a) Lepidoptera‐2 that was identified at the species level (*Melitaea deione*) by the 16S sequences and at the genus level by the COI sequences, and (b) Psocoptera‐2 that was identified at the genus level (*Myopsocus* sp.) by the 16S sequences and not identified by the COI sequences.

### 3.3 In vivo evaluation—Guano samples

#### 3.3.1 PCR verification and sequencing results

DNA amplification was relatively homogeneous between primer sets and pellet samples, except for Hex and Lep1. These both had amplification failures and nonspecific product amplifications. MiSeq sequencing produced a total of 9,190,350 reads. Removing reads with unexpected lengths excluded 0.52% (Epp‐degen) to 10.57% (Lep1) of the reads (and exceptionally 40.16% of the reads for Hex). Removing chimeras excluded 0.1% (Epp‐degen) to 6.42% (mlHCO) of the reads. Removing reads not shared by at least two PCR replicates excluded 0.3% (Epp‐degen) to 16.17% (Hex) of the reads. Finally, the remaining reads varied from 552,914 (Epp‐degen primer set) to 808,594 (Zeale primer set), with the primer set Hex remaining an outlier (368,121 reads).

#### 3.3.2 Detection of bat taxa

**FIGURE 5:**
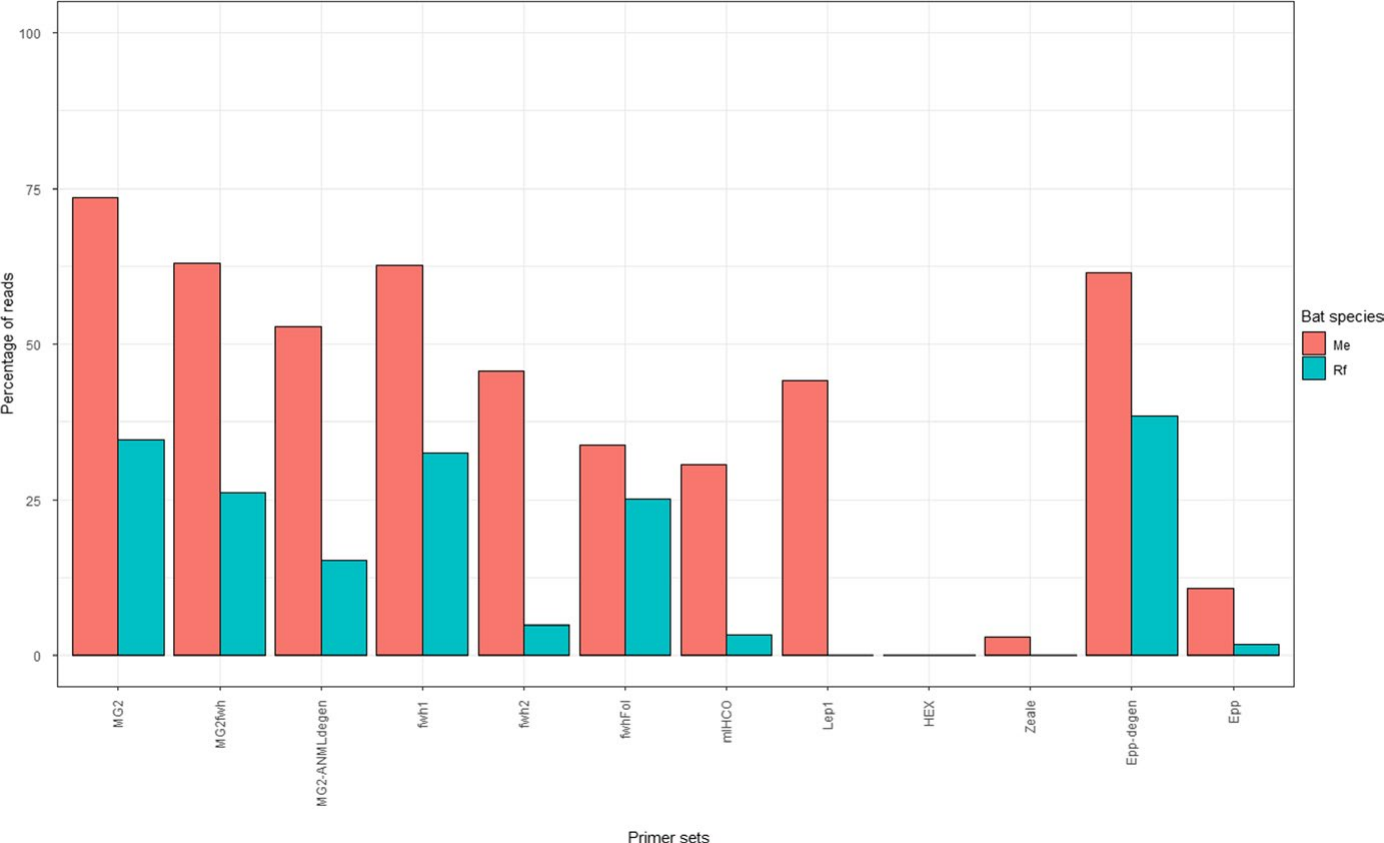
Percentage of bat reads in the guano samples from *Myotis emarginatus* in red (*N* = 11) and from *Rhinolophus ferrumequinum* in blue (*N* = 10) for each primer set

Two bat species were identified: *Rhinolophus ferrumequinum* and *Myotis emarginatus*. *Rhinolophus ferrumequinum* was predominant in ten samples (mean percentage of reads > 98.9% of all the Chiroptera reads), *M. emarginatus* was predominant in eleven samples (mean percentage of reads > 99.2%), and both species were found in high mixed proportions in one sample (mean percentage of *R. ferrumequinum* reads = 84.2% and mean percentage of *M. emarginatus* reads = 15.80%). This sample was discarded from further analyses. For eight primer sets and 12 samples, some reads of the wrong species (mean number of reads = 15, median = 2 reads; true species: mean = 3,760 reads, median = 2,961 reads) were also observed but in highly unbalanced proportions and only in some of the PCR replicates (55%), indicating very slight traces of cross‐contamination between pellets from different bat species in the colonies (contamination median number of reads < 0.05%; Table 3). Traces of DNA from another bat species, *Myotis myotis*, were also found in three samples by four primer sets (mean of 0.0001% of bat reads; Epp‐degen, fwh1, MG2, and MG2fwh). Moreover, some primer sets did not amplify any of the two bat species (Hex) or amplified only one bat species (Zeale, Lep1), whereas others amplified both bat species (Figure 5). However, the percentage of bat reads in *R. ferrumequinum* pellets was always lower than in *M. emarginatus* pellets, whichever primer set was considered (Figure 5). Finally, bat reads outnumbered arthropod reads for eight primer sets out of 12 in pellet samples from *M. emarginatus,* and for the 16S Epp‐degen primer set (Epp‐degen) in *R. ferrumequinum* pellet samples.

**TABLE 3:** Number of reads of the putatively wrong (number at the left) and right (number at the right) bat species in samples showing traces of reads of the wrong species. Ratios > 1% are written in bold

	Samples	Epp‐degen			fwh1			fwh2			fwhFol		
		PCR1	PCR2	PCR3	PCR1	PCR2	PCR3	PCR1	PCR2	PCR3	PCR1	PCR2	PCR3
Rf/Me	Me‐ANN‐0002	6/4330	13/9703	9/7250	74/11416	62/10914	63/13143	45/6342	28/6560	38/7137	**430/4875**	**382/4226**	**206/4343**
	Me‐ANN‐0004	2/5161	0/3370	1/7014	1/10315	6/10423	3/10762	0/5407	0/4950	0/5930	18/3715	5/2806	7/2742
	Me‐BEA‐0061	3/5066	0/3140	0/5699	0/6277	0/5893	0/4795	6/3638	1/2904	3/2258	0/2364	0/1088	1/1449
	Me‐BEA‐0082	**13/1430**	**7/1240**	**36/2783**	39/5294	21/3286	42/4160	0/3477	0/2092	0/3106	**118/1932**	**94/1453**	**100/1472**
	Me‐BEA‐0088	6/7408	3/6571	3/4814	6/10635	39/10779	45/8989	0/6941	0/7497	0/5964	**108/3161**	**42/4305**	**90/2767**
	Me‐SGE‐0010	1/7750	0/3138	0/7362	0/4713	0/2971	0/4473	0/2392	0/1966	0/2757	2/1406	5/2602	0/2143
	Me‐SGE‐0015	16/5271	6/2223	66/6961	2/2570	7/3481	5/2433	0/1915	0/5209	0/2620	**81/2030**	**55/2370**	**55/1945**
Me/Rf:	Rf‐ALL‐0001	2/1561	2/2043	2/3788	1/1520	18/3132	8/2033	**3/79**	**15/187**	**8/111**	1/1508	3/2978	5/1957
	Rf‐LES‐0003	0/3804	0/2965	0/2825	3/680	0/882	1/1100	**10/58**	**0/33**	**9/53**	0/2034	0/2103	0/1839
	Rf‐LES‐0004	0/3529	0/5397	0/1767	0/9093	0/6182	0/3280	0/1839	0/1117	0/1097	0/2737	0/6083	0/2721
	Rf‐SGE‐0001	0/1503	2/5719	1/3244	2/3577	18/3411	10/4960	**24/280**	**22/327**	**10/562**	9/3202	4/2385	8/1836
	Rf‐SGE‐0003	3/2751	2/1403	8/3053	**52/2669**	**0/1748**	**14/1072**	**140/304**	**0/248**	**78/139**	**48/2462**	**0/2075**	**28/1493**
	Mix‐BEA‐0002	**197/1868**	**111/1136**	**135/1169**	**841/2366**	**822/2610**	**792/2146**	**1317/239**	**1162/284**	**1043/177**	**555/2304**	**748/3827**	**417/1346**

		MG2			MG2ANML‐degen			MG2fwh			mlHCO		
		PCR1	PCR2	PCR3	PCR1	PCR2	PCR3	PCR1	PCR2	PCR3	PCR1	PCR2	PCR3
Rf/Me	Me‐ANN‐0002	16/8100	73/11908	47/9441	13/9335	20/7747	24/10514	91/12035	68/11445	36/9510	0/2292	0/2303	0/2024
	Me‐ANN‐0004	2/8300	2/6448	0/8725	0/7160	2/11378	0/11507	2/12072	1/6819	5/7782	0/2673	0/4785	0/3787
	Me‐BEA‐0061	7/6983	0/5113	0/6813	0/3721	0/3724	0/3251	3/4845	1/2384	3/3645	0/2163	3/1803	0/2138
	Me‐BEA‐0082	7/2668	2/2048	8/2970	5/2965	1/2815	6/2146	13/3332	7/2543	10/2922	0/2149	0/2394	0/1266
	Me‐BEA‐0088	8/10747	7/9836	14/7082	2/5670	4/8671	12/7289	17/8210	3/7934	8/6164	0/2158	0/3972	0/3551
	Me‐SGE‐0010	2/9093	0/6317	10/8806	0/3347	0/2227	0/2633	0/3013	1/3701	0/4660	0/3475	0/1810	0/2500
	Me‐SGE‐0015	34/7890	18/6096	67/8753	0/3015	0/3555	0/3381	1/2993	9/3440	3/2956	0/2556	0/2446	0/1326
Me/Rf:	Rf‐ALL‐0001	0/1708	18/2540	56/3467	0/624	0/383	0/295	0/1359	17/1684	3/1691	**17/69**	**10/22**	**13/20**
	Rf‐LES‐0003	0/2787	23/2175	0/1982	0/165	0/228	0/196	0/789	0/722	0/774	0/2	0/68	0/23
	Rf‐LES‐0004	0/5258	0/6837	0/2408	0/3549	0/3680	0/2972	0/3538	1/5278	2/3923	0/986	0/970	0/759
	Rf‐SGE‐0001	53/2351	44/5278	2/3460	2/1121	5/1344	0/792	15/2717	5/2032	30/1737	**14/144**	**0/273**	**2/373**
	Rf‐SGE‐0003	0/3788	118/2337	0/3768	0/740	0/433	0/298	0/1098	10/1095	0/821	0/200	0/286	0/130
	Mix‐BEA‐0002	**2148/2768**	**1425/1824**	**1286/1561**	**487/795**	**368/695**	**354/313**	**864/1403**	**884/1756**	**564/1074**	**863/77**	**744/101**	**363/20**

Note

Rf = *R. ferrumequinum*; Me = *M. emarginatus*.

#### 3.3.3 Detection of arthropod taxa

We found a negative effect of the amplicon length (*p* = .030) and a positive effect of the number of reads (*p* = .032) on the number of occurrences detected (Table 2). However, the number of reads was not significant (*p = *.397) when the primer set with the lowest number of reads (Hex) was excluded. The degeneracy level of the primers and the percentage of bat reads had no effect on the number of occurrences detected (*p* = .140 and *p* = .111, respectively).

**FIGURE 6:**
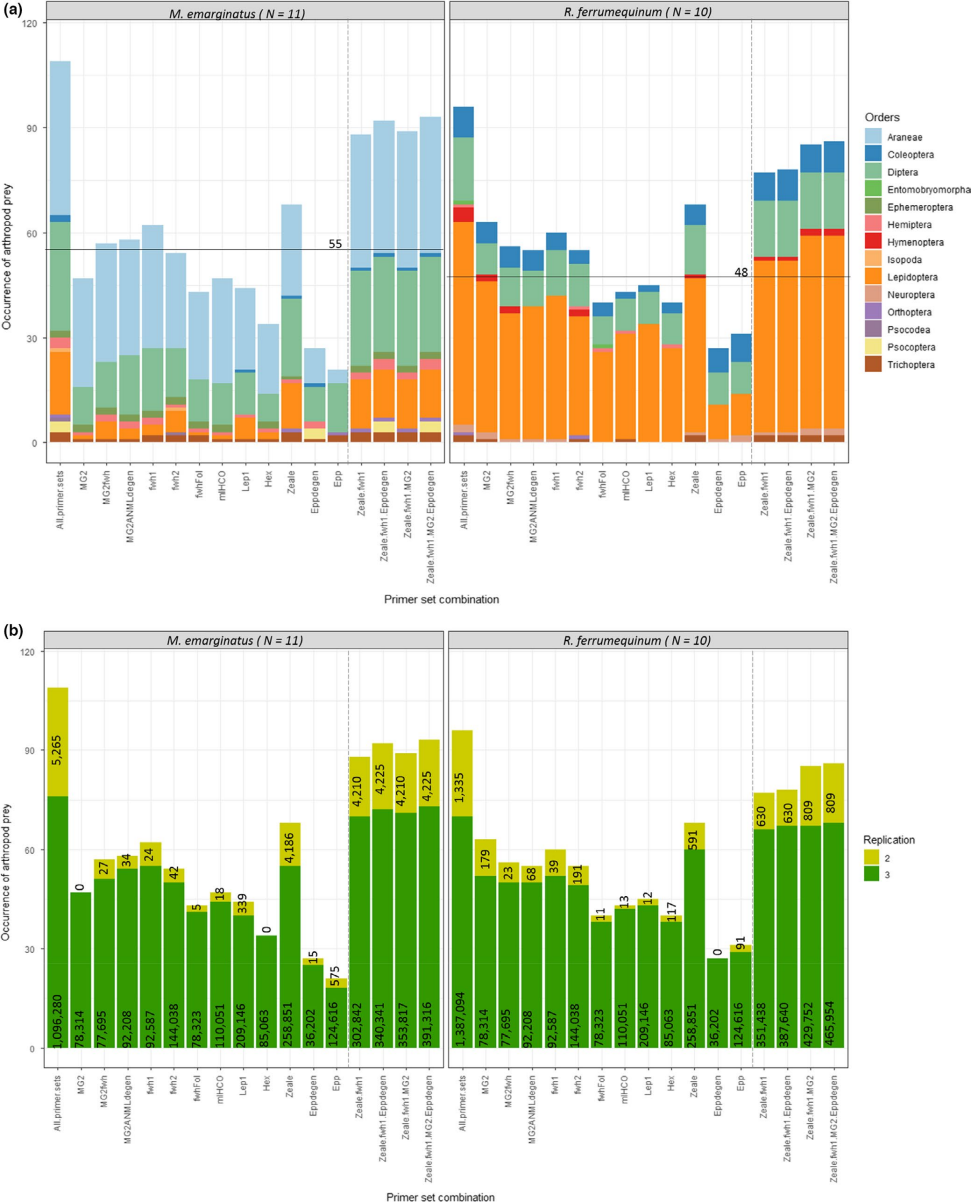
Occurrences of arthropod prey taxa detected in guano samples for each primer set and for four combinations of primer sets. Dashed lines separate the primer sets from the combinations of primer sets. *N* is the number of guano samples analyzed for each bat species. (a) Occurrences of arthropod orders. Black lines represent 50% of the occurrences (55 occurrences for *Myotis emarginatus* samples and 48 for *Rhinolophus ferrumequinum* samples). (b) Comparison of the number of occurrences of arthropod orders considering the reliability of the technical PCR replicates (dark green = occurrences validated in three PCRs out of three; light green = occurrences validated in two PCRs out of three) for each primer set on the one hand and for two to four primer sets in combination on the other hand. The latter include the two 16S primer sets and the COI primer sets that provided the best results in terms of occurrence of arthropod orders. Dashed lines separate the primer sets from the combinations of primer sets. Numbers correspond to the number of reads gathered for each class of occurrence validation (three PCRs out of three vs two PCRs out of three)

**FIGURE 7:**
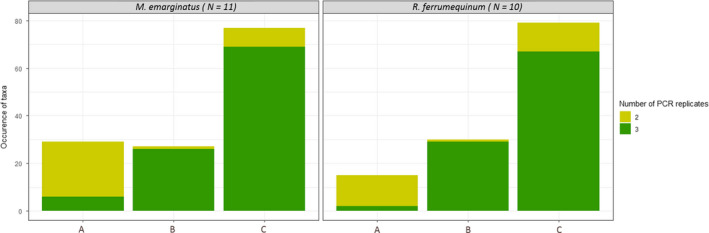
Reliability of the occurrences of prey taxa in *Myotis emarginatus* samples (*N* = 11) and *Rhinolophus ferrumequinum* (*N* = 10). The occurrences are grouped by bat species and ordered by level of reliability according to the repeatability between primer sets and PCR replicates: (a) not shared between the 12 primer sets (i.e., observed with only 1 of the 12 primer sets), (b) shared by at least one COI primer set and one 16S primer set, or (c) shared by at least two COI primer sets. Number of positive PCR replicates: “2” (light green) = occurrence in two PCRs out of three; and “3” (green) = occurrence in three PCRs out of three

Taken together, our results showed that the 12 primer sets allowed the identification of 96 taxa (from 10 orders, 32 families, 56 genera, and 60 species) in *R. ferrumequinum* samples and 109 taxa (from 11 orders, 34 families, 63 genera, and 65 species) in *M. emarginatus* samples. The 16S primer sets revealed about 30% and 20% of the occurrences in *R. ferrumequinum* and *M. emarginatus* samples, respectively (Figure 6a). Six and four of the ten COI primer sets allowed for the detection of at least 50% of the occurrences of prey items in *R. ferrumequinum* and *M. emarginatus* samples, respectively (Figure 6a). The other COI primer sets revealed between 31.2% and 49.5% of the total number of occurrences. The Zeale primer set revealed the highest number of arthropod occurrences for both bat species (*N* = 68, 62% of *R. ferrumequinum* occurrences; and *N* = 68, 71% of *M. emarginatus* occurrences); however, this primer set was unable to amplify and identify bat species. The optimal primer set that amplified bat DNA and provided the highest number of arthropod occurrences for both bat species was fwh1 (number of occurrence_(_ *_R. ferrumequinum)_* = 60; number of occurrence_(_ *_M. emarginatus)_* = 62) although MG2 was slightly better than fwh1 for *R. ferrumequinum* samples (number of occurrence_(_ *_R. ferrumequinum)_* = 63). The combination of two to four primer sets (Epp‐degen, fwh1, MG2, and Zeale) allowed for a gain of nine to 18 occurrences in *R. ferrumequinum* samples (up to 89.58% of the occurrences detected by combining all primer sets) and of 20–25 occurrences in *M. emarginatus* samples (up to 85.32% of the occurrences detected by combining all primer sets) (Figure 6a). However, about one‐third of all the occurrences corresponded to potentially unreliable amplifications (amplifications in two PCRs out of three and/or amplifications by only one primer set) associated with a low number of reads (Figures 6b and 7). Indeed, we observed that 79.3% (*M. emarginatus*) and 86.7% (*R. ferrumequinum*) of the occurrences found by only one of the primer sets out of the 12 were not reproducible across the three PCR replicates (Figure 7). In *R. ferrumequinum* samples, these potentially unreliable occurrences represented a very low number of reads whichever primer set was considered (median < 39 reads). In *M. emarginatus* samples, these potentially unreliable occurrences also represented a very low number of reads ranging from 5 to 575 (exceptionally 4,186 for Zeale) for all primer sets (Figure 6b). The maximum number of reads for the potentially unreliable occurrences was lower in *R. ferrumequinum* than in *M. emarginatus* samples (591 reads, Zeale; Figure 6b).

### 3.4 Multicriteria evaluation of primer sets

Based on the scores of mean penalty and theoretical amplification success provided by *PrimerMiner*, we identified seven appropriate primer sets (MG2, MG2fwh, MG2ANML‐degen, fwh1, fwhFol, Hex, and Epp‐degen), one intermediate (fwh2), and four inefficient primer sets (Lep1, mlHCO, Zeale, and Epp) (Table 4). The number of detected taxa (bat and arthropod) and the taxonomic resolution of the latter indicated that seven primer sets were equivalent considering mock community results: MG2, MG2fwh, MG2ANML‐degen, fwh1, fwh2, fwhFol, and mlHCO (Table 3). Considering guano results, Zeale and fwh1 had the best scores. However, Zeale did not amplify bat DNA, which is critical when studying environmental DNA from feces that can potentially originate from different predator species. Overall, the best primer set for our study was therefore fwh1 (Table 4).

**TABLE 4:** Multicriteria table indicating the score of each primer set for the three assessment steps performed

Analyses	Criteria	MG2	MG2fwh	MG2ANML‐degen	fwh1	fwh2	fwhFol	Lep1	mlHCO	Hex	Zeale	Epp‐degen	Epp	Score details		
In silico	Mean amplification success	3	3	3	3	2	3	1	1	3	1	3	1	≥100	]50:100[	≥50
	Mean penalty	3	3	3	3	2	3	1	1	3	1	3	1	≤50	]50:80[	≥80
In vivo—mock communities	Bat identification	3	3	3	3	3	3	1	3	1	1	3	2	0 PCR/3	2 PCRs/3	3 PCRs/3
	Arthropod taxa detection success	3	3	3	3	3	3	1	3	3	1	3	2	≤80%	]80%: 90%[	≥90%
	Identification at the species level	3	3	3	3	3	3	2	3	3	2	1	1	≤10 taxa	]10:15[	≥15
In vivo—guano	Bat identification	3	3	3	3	3	3	2	3	1	1	3	3	0	1 sp	2 sp
	Occurrence of prey taxa	2	2	2	3	2	1	1	1	1	3	1	1	≤100	]100:130[	≥130
	Number of orders	2	2	2	2	3	2	2	2	2	3	2	2	≤5	]5:10[	≥10
	Number of families	2	3	2	3	3	1	2	1	1	3	1	1	≤25	]25:30[	≥30
	Number of genus	2	2	2	3	2	2	1	2	1	3	1	1	≤50	]50:70[	≥70
	Number of species	2	2	2	3	2	1	2	1	1	3	1	1	≤60	]60:80[	≥80
	Cumulative score	28	29	28	32	28	25	16	21	20	22	22	16			

Note

Scores range from the less efficient (“1” represented in red) to the most efficient (“3” represented in green).

## 4 DISCUSSION

During the most recent decade, eDNA metabarcoding has proven to be a promising approach for characterizing biodiversity in a broad array of contexts (Bohmann et al., 2014). The accuracy and completeness of metabarcoding results are critical from a fundamental point of view, and because these data could potentially be used to inform environmental management strategies. Previous metabarcoding studies highlighted how the choice of primer sets may influence the detection of particular arthropod species in diet analyses, and in turn the interpretation of trophic ecology (e.g., Esnaola et al., 2018).

### 4.1 The need for primers that identify predator species and discard contaminated samples

The need for identifying predators in insectivorous diet studies strongly depends on the sampling scheme and on the ecology of the targeted organisms. Such identification is especially important when using environmental samples, such as fecal samples, as it ensures that samples belong to the species of interest and it eliminates erroneous assignation of prey (Ware et al., 2019). For example, the metabarcoding study performed by Forin‐Wiart et al. (2018) on cat fecal samples revealed that 2.4% of these belonged to another predator species despite a preselection of samples using qPCR screening. Similarly, Biffi et al. (2017) sequenced 560 feces assumed to belong to the Pyrenean Desman. They showed that, in fact, 170 samples belonged to 25 other predator species (birds and mammals). In the particular case of insectivorous bats, the identification of bat species from guano is critical when guano is collected in roosts, especially when bat colonies are known to be mixed. It is potentially less important when guano is retrieved from trapped bats, or from monospecific bat colonies. However, even colonies supposed to be monospecific can be shared by cryptic bat species (Filippi‐Codaccioni et al., 2018) or other insectivorous species (e.g., birds). Furthermore, we have shown that some of the guano sampled in the roosts of mixed colonies (*R. ferrumequinum*/*M. emarginatus*) was contaminated with excreta belonging to other bat species, including *M. myotis*, for which a few individuals were also known to be present in the studied colony. Hence, future diet analyses should allow for the simultaneous identification of bat species and their prey, to reveal the presence of unexpected species in the roosts studied, and to discard guano that would be contaminated by the DNA of multiple predators. It is thus particularly important to ensure that primer sets are able to identify all bat species potentially present in the roosts. Here, we have shown that some primer sets did not amplify bat species at all and that others could provide bat identification only for some of the bat species of interest (one out of three). We performed a posteriori in silico analyses to test the efficiency of our primer sets on four bat families (Molossidae, Mormoopidae, Rhinolophidae, and Vespertilionidae). Using this larger spectrum of Chiropteran families and species, our results tended to confirm the contrasting pattern of bat amplification observed between our primer sets, albeit with low power due to a low numbers of OTUs (see Appendix S1).

The simultaneous identification of predator and prey is often avoided in diet analyses as an elevated predator amplification is expected to dampen that of prey (Pompanon et al., 2012; Vestheim & Jarman, 2008). Other approaches used to be applied to ensure that the samples belong to the relevant species, including a separate diagnostic PCR that is specific to the expected species, and/or Sanger sequencing that only reveals the major DNA sequence of the sample (Bohmann et al., 2011; Forin‐Wiart et al., 2018). These alternatives add a supplementary step to the metabarcoding process, thereby increasing the time and financial cost of the analyses. Most importantly, they do not reveal the presence of nontargeted predator species, or the rate of between‐species contamination of samples. As such, they do not facilitate the rejection of contaminated samples. In this study, we have shown that the simultaneous predator amplification by metabarcoding does not necessarily lead to a drop in prey detection. Our results revealed that there is no effect of the percentage of bat reads on the percentage of arthropod taxa detected in mock communities, nor on the number of arthropod occurrences in guano samples. This corroborates the results of Galan et al. (2018) which showed well‐balanced proportions of reads for 16 bat species and their prey using a previous version of the MG2 primer set used here. We therefore recommend the simultaneous identification of predator species when working on environmental fecal samples.

### 4.2 Choosing between the “16S + COI” and “COI‐alone” strategies

In this study, we compared primers designed from two mitochondrial genes that are frequently used in eDNA metabarcoding studies of animals (Deagle et al., 2014): the COI and 16S genes. Our results showed that the 16S primer sets were always less efficient and had lower levels of taxonomic resolution than most of the COI primer sets tested. This was surprising, as, for example, the 16S Epp‐degen was identified as one of the best primer sets from the in silico analysis. Moreover we expected a high amplification success for this primer set due to its short amplicon length and high level of degeneration. In consequence, it is likely that the poor performance of the 16S primers revealed in the guano analyses is due to the paucity of reference sequences in the 16S database; an issue which has been emphasized in previous studies (Clarke et al., 2014; Elbrecht et al., 2016; Marquina et al., 2019). This difference in diversity between COI and 16S reference databases could explain the differences in the number of taxa which could be assigned to genera or species. Lack of reference sequences could also bias results when analyzing short‐length amplicons. Indeed, as there are less reference sequences, the risk of obtaining an identification with strong confidence levels for the wrong taxa is potentially higher. For example, the mock community analyses showed that Lepidoptera‐2 was identified to the species level by the 16S sequences (only one species *Melitaea deione* was available in the database) and to the genus level by the COI sequences because five different *Melitaea* species occurred in the database with identical similarities to the query sequence. Also, Psocoptera‐2 was identified to the genus level (*Myopsocus* sp.) by the 16S sequences and not identified by the COI sequences because none of the 43 *Myopsocus* vouchers with 100% identity to our sequences were identified to the species level in the Bold Species Level Barcode Records database. Moreover, this lack of reference sequences also increases the possibility that 16S affiliations are different from COI ones, therefore leading to a false increase in species richness when combining both genes, as it was observed in this study for mock community analyses (Trichoptera‐1 identified as *Stenophylax vibex* for COI primer sets and *Anabolia bimaculata* for 16S primer sets). Therefore, the choice of the marker in metabarcoding studies should be strongly guided by the comprehensiveness of the reference databases available. We advocate for the use of the COI gene in animal metabarcoding studies because of its extensive database. However the 16S gene can be used if a sequence database of local species of interest is specifically created (Elbrecht et al., 2016).

### 4.3 How many COI primer sets should be used?

To counter the negative effects of primer biases, two main strategies have recently been proposed considering the COI gene. Corse et al. (2019) advocated for the use of multiple primer sets as a solution to describe species diversity in fine detail**,** to reveal greater diversity, and to decrease false‐negative results. However, their in silico analysis showed that the three primer sets considered were not degenerate enough to correctly amplify a large spectrum of prey. Nevertheless, the negative effects of primer biases can be reduced by incorporating primer degeneracy and by carefully choosing primer sets suited for the targeted ecosystems and taxonomic groups of interest (Elbrecht et al., 2019). In our study, no primer set alone was able to detect all arthropod occurrences in the guano samples. This was also the case for the very recent primer set comparison of Elbrecht et al. (2019) based on a Malaise trap capture. At first glance, our results showed that combining up to four primer sets allowed for a gain of between 8.5% (Zeale + fwh1) and 17% (Zeale + fwh1 + MG2 + Epp‐degen) of arthropod occurrences in *R. ferrumequinum* samples and between 20.8% (Zeale + fwh1) and 26% (Zeale + fwh1 + MG2 + Epp‐degen) of arthropod occurrences in *M. emarginatus* samples. We might first hypothesize that this gain might at least partly result from the increased number of technical replicates associated with the combination of primer sets, independently of the primer sets' characteristics or the taxonomic origin of the prey. This hypothesis is reinforced by the gain in mainly less repeatable occurrences (between primer sets and PCR replicates) which are based on a low number of reads when combining several primer sets (see light green bar in Figure 6). It would thus be interesting to experimentally test the hypothesis of the effect of the number of PCR replicates on prey detection. Alternatively, our results could advocate for the use of multiple primers. Deciphering between the “one‐locus” versus “multilocus” strategies should hence rely on the trade‐off between the completeness of the results gathered on the one hand and the costs of combining several primers on the other. However, a mean of 23.8% (*R. ferrumequinum*) and 31.6% (*M. emarginatus*) of these occurrences were characterized by the amplification of only two replicates out of three and a small number of reads (except Zeale > 4,000 reads in *M. emarginatus* samples). These particular occurrences, which were mostly amplified by a single primer set out of twelve, might be the reason why the plateau of taxa occurrence could not be reached with the combination of a reasonable number of primer sets. These less repeatable occurrences may not concern specific taxonomic groups with particular primer sets but rather could be due to the weak biomass of prey (low DNA quantity), traces of old meals (very degraded DNA, as observed with the Hemiptera‐1 of our mock community), traces of secondary predation (e.g., meals of Araneae, the more frequent order in the *M. emarginatus* diet), or environmental contaminations potentially from other bat or insectivorous species. 79.3% (*M. emarginatus*) and 86.7% (*R. ferrumequinum*) of the occurrences that were revealed by a single primer set, and that could therefore be interesting to recover by combining several primer sets, were less repeatable (i.e., revealed by a single primer set + only two positive PCRs out of three for these taxa + small number of reads). These results bring new insights into the real benefit of attempting to recover all taxonomic occurrences, in the case where one‐third of them were less repeatable with regard to PCR replication, and not replicable between primer sets. Therefore, the use of a single primer set following the characteristics described below may well be sufficient.

### 4.4 What is the best COI primer set for characterizing insectivorous diets?

Previous studies have shown that in silico and in vivo tests were complementary and critical for assessing the performance of primers (Alberdi et al., 2018; Corse et al., 2019; Elbrecht et al., 2019). Here, the combination of in silico, mock community, and guano analyses enabled us to reveal the strengths and weaknesses of 12 primer sets for a large spectrum of taxa. DNA quality did not influence in silico and mock community analyses, so that amplicon length had no significant effect on the number of arthropod taxa detected, while the degeneracy level of the primers had a major positive effect on it as it minimized mismatches between primers and sequences from diverse taxonomic assemblages (Braukmann et al., 2019).

However, amplicon length was the most important factor influencing the success of arthropod detection when analyzing DNA from guano samples. Amplicons that were too long (>313 bp), such as Hex and fwhFol, were less efficient. This result could be explained by the low proportion of large size DNA fragments in degraded samples. Therefore, our results strongly support the use of short‐length amplicons and degenerate primers to maximize biodiversity coverage in metabarcoding analyses (Elbrecht et al., 2019; Elbrecht & Leese, 2017; Galan et al., 2018; Vamos et al., 2017).

In previous studies, the Zeale primer set seemed well suited for detecting Lepidoptera and Diptera because of its high taxonomic coverage for these orders (Clarke et al., 2014; Zeale et al., 2011). As the diet of European bats is mainly dominated by Lepidoptera and Diptera (Alberdi, Razgour, et al., 2019), the Zeale primer set remains one of the most used in insectivorous bat diet studies (e.g., Aizpurua et al., 2018; Andriollo, Gillet, Michaux, & Ruedi, 2019; Clare, Symondson, & Fenton, 2014; Vesterinen, Puisto, Blomberg, & Lilley, 2018). In our study, the Zeale primer set (Zeale et al., 2011) showed important amplification failures during in silico and mock community analyses, but not in guano analyses. Low taxon recovery of the Zeale primer set had previously been observed for terrestrial arthropods (Brandon‐Mong et al., 2015; Clarke et al., 2014; Elbrecht et al., 2019; Esnaola et al., 2018), especially when facing a large spectrum of arthropods, as shown for the insectivorous Pyrenean Desman (Esnaola et al., 2018) or for some insectivorous bat species (Jusino et al., 2018). Moreover, it is important to keep in mind that the Zeale primer set does not allow the identification of bat species. We thus do not recommend it in eDNA studies, especially when several insectivorous predator species may share the same sites. Otherwise, it would be necessary to add another primer set for predator detection and identification (e.g.,Biffi et al., 2017; Vesterinen et al., 2018). Lastly, numerous taxa detected with the Zeale primer set were (a) not observed with other primer sets, as noted by Elbrecht et al. (2019) who underlined the putative presence of false‐positive results, or (b) weakly reliable considering PCR replication results. Therefore, previous studies of insectivorous (bat) diet based on this primer set should be interpreted with some caution as results may be affected by the potential biases described above.

Here, we demonstrated that fwh1 was the best primer set identified to simultaneously characterize the diet of *R. ferrumequinum* and *M. emarginatus* from guano samples collected under mixed roosts (Vamos et al., 2017) (Table 4). However, we have also shown that the efficiency of the primer sets, in terms of arthropod detection, may vary between the two bat species studied here. We therefore recommend that a few primer sets should be tested on a representative subset of eDNA samples before undergoing large‐scale metabarcoding studies of diet for the first time. Such preliminary analyses should help to determine the most suitable primer set with regard to the sampling scheme and the biological model targeted.

## 5 CONCLUSION

Our study confirms the importance of combining in silico, mock community, and field sample analyses to determine the benefits and the limits of potential primer sets before conducting research based on metabarcoding. Here, this three‐step assessment of primer performance confirmed that primer success was determined by amplicon length, base degeneracy level, and how complete reference databases are. Our work also emphasized that the identification of the best primer sets for insectivorous diet studies was not only highly dependent on the objective and financial resources of the study, but it also varied depending on the sampling protocols and constraints that might impact DNA quality and make the identification of predator species necessary. Finally, our results emphasized the presence of potential unreliable occurrences of taxa (detected by a single primer set out of twelve + amplification of only 2 PCRs out of three + small number of reads). Instead of combining numerous primer sets to recover these taxa, we suggest instead to increase the number of PCR replicates. In conclusion, we advocate for the use of multicriteria assessments that summarize all the information required to evaluate any primer sets' performance. This analytical framework can easily be adapted to other metabarcoding studies of predator diet.

## CONFLICT OF INTEREST

The authors declare no conflict of interest.

## AUTHOR CONTRIBUTION

**Orianne Tournayre:** Conceptualization (equal); data curation (equal); formal analysis (equal); investigation (equal); methodology (equal); validation (equal); visualization (equal); writing – original draft (equal); writing – review & editing (equal). **Maxime Leuchtmann:** Funding acquisition (equal); investigation (equal); project administration (equal); resources (equal); writing – review & editing (equal). **Ondine Filippi‐Codaccioni:** Investigation (equal); project administration (equal); resources (equal); writing – review & editing (equal). **Marine Trillat:** Formal analysis (equal); writing – review & editing (equal). **Sylvain Piry:** Formal analysis (equal); writing – review & editing (equal). **Dominique Pontier:** Conceptualization (equal); funding acquisition (equal); project administration (equal); resources (equal); supervision (equal); Writing‐original draft (equal); writing – review & editing (equal). **Nathalie Charbonnel:** Conceptualization (equal); funding acquisition (equal); project administration (equal); resources (equal); supervision (equal); writing – original draft (equal); writing – review & editing (equal). **Maxime Galan:** Conceptualization (equal); data curation (equal); formal analysis (equal); investigation (equal); methodology (equal); resources (equal); validation (equal); visualization (equal); writing – original draft (equal); writing – review & editing (equal).

## Supporting information

Figure S1

Appendix S1

## Data Availability

Supplementary data deposited in Dryad (https://doi.org/10.5061/dryad.4v9n227) include: (a) raw sequence reads (fastq format), (b) raw abundance tables and (c) reference sequences of the 33 arthropod taxa and the bat used in the mock communities, and (d) the following appendix, tables and figures: Appendix D1. Rules used to determine the final identifications of OTUs in guano samples. Table D1. Number of COI and 16S OTUs and sequences, obtained with sequences from BOLD and NCBI databases, respectively. Table D2. Information about the samples, the laboratory controls and the technical replicates. Table D3. Mixes of dephasing primers used in PCR1 of the 2‐step PCR including heterogeneity spacers and Illumina sequencing primer sequences. Table D4. Information about the sequencing libraries derived from the samples, the laboratory controls and the technical replicates. Error‐proof indexes for high throughput sequencing were created by Martin (2019). Table D5. Number of reads of the genuine and reference sequences of the two mock communities, for each PCR replicate and each primer set. Table D6. Examples of affiliation errors for several primer sets in reference databases. Table D7. Mean penalty scores obtained for each arthropod order (OTUs > 100) with each primer set using the PrimerMiner program. Table D8. In silico amplification success obtained for each arthropod order (OTUs > 100) and primer set using the PrimerMiner program. Table D9. Objectives and impact of the pre‐process and FROGS pipelines on the number of reads. 'Complete run ' indicates that the run included samples other than those of the two mock communities. ¥ = genuine sequences, † = identity >97% and coverage >90%, ‡ identity <97% and/or coverage <90%. Table D10. Molecular identification of the taxa in the mock communities obtained using the minibarcode sequences of each specimen, respectively for each primer set and the Folmer region. '<97%' indicated a percentage of identity lower than 97%. Figure D1. Consensus sequence alignment of 21 arthropod orders using the PrimerMiner R package. Figure D2. PCR products of the mock communities (left column) and guano samples (right column) on agarose gel. Figure D3. Picture of the DNA extractions on agarose gel for the 35 samples used in the two mock communities.
