# Supplementary material for: In silico and empirical evaluation of twelve metabarcoding primer sets for insectivorous diet analyses
Source: Ecol Evol. 2020 May 21;10(13):6310–32. doi: 10.1002/ece3.6362 (PMC7381572; doi:10.1002/ece3.6362)
Supplement: Supplementary file 2 — Appendix S1 [file ECE3-10-6310-s002.docx]

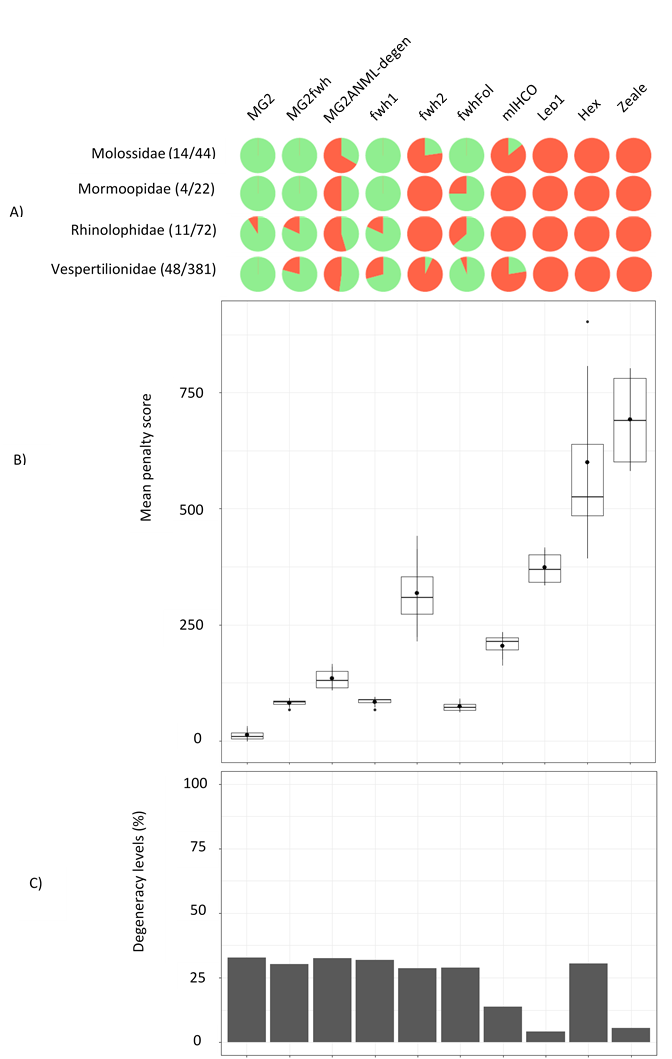


Appendix S1. *In silico* evaluation of COI primer sets on a wide range of phylogenetically diverse bats using *PrimerMiner*. We analysed four bat families for which mitogenomes were available. Mitogenomes permit the addition of primers outside the 658bp Folmer region (MG2-LCO1490, fwhF1, LepF1, HCO2198 and Fol-degen-Rev) in this evaluation. The numbers in parentheses represent the number of OTUs for which we have information outside of the Folmer region / the total number of OTUs for the bat family, respectively. A) Primer set performance is shown for each family using pie charts, with green and red colours representing success and failure of amplification, respectively. Success of amplification corresponded to *PrimerMiner* mean penalty score < 120 and amplification failure to a mean penalty score >= 120. B) Boxplots of the median of *PrimerMiner* mean penalty scores over all bat families and for each primer set, with mean values represented by a circle within boxplots. C) Percentage of degeneracy level of each primer set. 16S primer sets are excluded from this comparison due to the lack of bat sequences in NCBI.
